# Medicaid prescription cap policies and exemptions for medications for opioid use disorder: A process and content analysis

**DOI:** 10.1093/haschl/qxaf203

**Published:** 2025-10-27

**Authors:** Jaclyn M W Hughto, Stephanie Vento, David R Pletta, Madeline Noh, Theresa I Shireman, Christopher M Santostefano, Landon D Hughes, Lisa Peterson, Emma Seymour, Elizabeth G Stettenbauer, Patience M Dow

**Affiliations:** Department of Behavioral and Social Sciences, Brown University School of Public Health, Providence, RI 02903, United States; Department of Epidemiology, Brown University School of Public Health, Providence, RI 02903, United States; Center for Health Promotion and Health Equity, Brown University School of Public Health, Providence, RI 02903, United States; Center for Alcohol and Addiction Studies, Brown University School of Public Health, Providence, RI 02903, United States; Center for Health Promotion and Health Equity, Brown University School of Public Health, Providence, RI 02903, United States; School of Law and Criminology, Maynooth University, Co. Kildare W23 F2H6, Ireland; Center for Health Promotion and Health Equity, Brown University School of Public Health, Providence, RI 02903, United States; Center for Health Promotion and Health Equity, Brown University School of Public Health, Providence, RI 02903, United States; Department of Health Services, Policy, and Practice, Brown University School of Public Health, Providence, RI 02903, United States; Center for Gerontology and Healthcare Research, Brown University School of Public Health, Providence, RI 02903, United States; Department of Health Services, Policy, and Practice, Brown University School of Public Health, Providence, RI 02903, United States; Center for Gerontology and Healthcare Research, Brown University School of Public Health, Providence, RI 02903, United States; Department of Epidemiology, Harvard T.H. Chan School of Public Health, Boston, MA 02115, United States; Department of Population Medicine, Harvard Pilgrim Health Care Institute, Boston, MA 02215, United States; VICTA, LLC, Providence, RI 02907, United States; Department of Health Services, Policy, and Practice, Brown University School of Public Health, Providence, RI 02903, United States; Department of Health Services, Policy, and Practice, Brown University School of Public Health, Providence, RI 02903, United States; Center for Alcohol and Addiction Studies, Brown University School of Public Health, Providence, RI 02903, United States; Department of Health Services, Policy, and Practice, Brown University School of Public Health, Providence, RI 02903, United States; Center for Gerontology and Healthcare Research, Brown University School of Public Health, Providence, RI 02903, United States

**Keywords:** Medicaid, prescription caps, medications for opioid use disorder (MOUD), content analysis

## Abstract

**Introduction:**

Some state Medicaid programs place a cap on the monthly number of covered prescription fills, including medications for opioid use disorder (MOUD)–the most effective OUD treatments.

**Methods:**

Between 2023 and 2024, we employed a quasi-systematic 3-step process (online search, survey of Medicaid experts, request-for-information) to identify contemporary Medicaid cap policy information and conducted a content analysis of cap policies.

**Results:**

Of the 12 states with contemporary prescription cap policies, 9 operated general caps and 3 operated caps for controlled substances. Across states, caps ranged from 3-6 monthly prescriptions. All states had exemptions based on beneficiary characteristics (eg, age, health conditions) or medication type (eg, contraceptives, antipsychotics), 6 of which had cap override policies, and 5 had MOUD-specific exemptions.

**Conclusion:**

Our search identified a dearth of publicly accessible, contemporary information on Medicaid cap policies, indicating a potential barrier to beneficiaries' understanding their prescription drug benefits. Further, although all states provided some type of policy carveout, half of the Medicaid programs operating caps did not exempt MOUD, which may negatively impact access to medically necessary medications for Medicaid beneficiaries with OUD.

## Teaser

Prescription drug limits or caps in state Medicaid programs are intended to control costs, but they can also restrict access to life-saving treatments such as medications for opioid use disorder. This study examines how states approach prescription cap policies, with a focus on whether medications for opioid use disorder are exempted from prescription caps. We analyzed state policy documents and data gathered from state Medicaid experts, identifying substantial variation in both the scope of caps policies and exemptions for medications for opioid use disorder. These differences have critical implications: in states where caps are stricter and exemptions are limited, patients may face greater barriers to initiating and continuing treatment. These findings underscore the urgent need for Medicaid programs to align prescription policies with public health priorities, including efforts to address the opioid epidemic. Clear, consistent exemptions for addiction treatment can reduce overdose risk and support recovery, while broader reforms could improve equity in access to medications for low-income populations. This research offers timely evidence on the nature of cap policies across Medicaid programs. Findings from this study, together with research linking Medicaid caps to reduced MOUD prescriptions and increased hospitalizations, may prove useful to state and federal policymakers as they consider whether and how to implement cost-containment strategies in the wake of federal cuts to Medicaid.

Teaser text Prescription drug limits or caps in state Medicaid programs are intended to control costs, but they can also restrict access to life-saving treatments such as medications for opioid use disorder. This study examines how states approach prescription cap policies, with a focus on whether medications for opioid use disorder are exempted from prescription caps. We analyzed state policy documents and data gathered from state Medicaid experts, identifying substantial variation in both the scope of caps policies and exemptions for medications for opioid use disorder. These differences have critical implications: in states where caps are stricter and exemptions are limited, patients may face greater barriers to initiating and continuing treatment. These findings underscore the urgent need for Medicaid programs to align prescription policies with public health priorities, including efforts to address the opioid epidemic. Clear, consistent exemptions for addiction treatment can reduce overdose risk and support recovery, while broader reforms could improve equity in access to medications for low-income populations. This research offers timely evidence on the nature of cap policies across Medicaid programs. Findings from this study, together with research linking Medicaid caps to reduced MOUD prescriptions and increased hospitalizations, may prove useful to state and federal policymakers as they consider whether and how to implement cost in the wake of federal cuts to Medicaid.

## Introduction

In the United States (U.S.), an estimated 5.7 million people 12 years of age and older met the criteria for an opioid use disorder (OUD) in 2023.^1^ Despite medications for opioid use disorder (MOUD) being the gold standard treatment for OUD^2,3^ and buprenorphine and methadone being protective against fatal overdoses,^4^ only 18.3% of those with OUD were treated with one or more forms of MOUD in the past year.^1^ Given the high rates of unemployment and related lower incomes among people with OUD,^3,5,6^ access to prescription health insurance benefits through state Medicaid programs plays a vital role in facilitating MOUD access.^7^

Medicaid is the single largest payer for OUD treatment services in the United States, insuring approximately 40% of non-elderly adults with OUD.^8,9^ While eligibility for Medicaid varies on a state-by-state basis, states that expanded coverage to adults under the Affordable Care Act in 2010 generally offer insurance to those with an income at or below 133% of the federal poverty level.^10^ Medicaid coverage typically includes prescription medications, including MOUD, at low or no copays; however, the out-of-pocket cost for MOUD can still vary significantly by state. For example, one study found a 17% reduction in substance use or behavioral health outpatient services and a 50% reduction in inpatient visits among Medicaid beneficiaries with copays vs no copays,^11^ highlighting the significant role that Medicaid coverage policies can play in the uptake of substance use treatment, including MOUD.

Due to the perpetual strain on state budgets, some Medicaid programs employ strategies aimed at containing prescription costs.^12^ One approach is to limit the number of covered prescriptions a Medicaid beneficiary can fill within a certain timeframe (commonly called prescription cap policies), typically per month.^7,13,14^ Prescription cap policies have the potential to exacerbate the opioid and overdose epidemics, as Medicaid beneficiaries with OUD and additional chronic conditions that require prescription medication may need to decide between receiving MOUD or other medications.^7,15,16^ Although the prescription cap policies' tradeoffs and impact on morbidity and mortality have been documented in other subsets of the Medicaid population,^17,18^ the role of prescription cap policies in fueling OUD-related morbidity and mortality among the more than 1.8 million Medicaid beneficiaries treated for an OUD is understudied.^19^

To ensure access to effective treatments for Medicaid beneficiaries with OUD, it is essential to understand overall contemporary cap policies and those specifically related to MOUD. Prior research on general cap policies indicates heterogeneity across state Medicaid programs.^14^ For instance, 2019 data from the Kaiser Family Foundation (KFF) showed that Alabama Medicaid covered up to 5 prescription medications per month, of which 4 could be brand-name, whereas Oklahoma Medicaid covered up to 6 prescriptions per month, of which 2 could be brand-name.^13^ While the 2019 KFF cap policy inventory is a useful resource, like the evolving opioid epidemic, Medicaid policies change over time, and 2019 data may not necessarily reflect the contemporary policies impacting Medicaid beneficiaries with OUD. A comprehensive analysis of the state Medicaid prescription cap policies is needed to understand the cap policy landscape generally and as it relates to MOUD coverage. Such data can inform longitudinal analyses exploring the impact of cap policies on MOUD access and related morbidity and mortality outcomes.

In this mixed-methods study, we employed a multi-step approach to (1) capture where and to what extent information on general and MOUD-specific prescription cap policy information could be readily identified, and (2) comprehensively summarize existing Medicaid prescription cap policies by state Medicaid program. Insights from the current study have the potential to inform policy-related research as well as serve as a resource to prescribers, pharmacists, and Medicaid beneficiaries who may be affected by these policies.

## Data and methods

Between September 2023 and September 2024, we conducted a two-phase approach to search for the most contemporary Medicaid cap policy coverage information (Phase 1) and analyze existing policies (Phase 2). This study does not meet the definition of human subjects research as the data collected and analyzed pertained to government policies; thus, it was exempt from Institutional Review Board review.

### Phase 1: multi-step process to identify Medicaid cap policies

The objectives of Phase 1 were to identify the existence and nature of cap policies and to qualitatively and quantitatively document the ease or difficulty of identifying this policy information. Phase 1 began with an online search to identify Medicaid prescription cap policy information (Step 1), followed by a brief survey of state Medicaid policy experts (Step 2). Phase 1 concluded with a formal request for information (Step 3) from states in which contemporary policy information of interest could not be identified via the previous two steps. Using the 2019 KFF Medicaid cap policy inventory, we narrowed our initial search process to Alabama, Arkansas, Florida, Georgia, Illinois, Kansas, Louisiana, Mississippi, Oklahoma, Tennessee, Texas, and Wisconsin. California was excluded as the state Medicaid program eliminated the cap policy in 2020. We then conducted an internet search of the gray literature, reviewed the peer-reviewed literature, and consulted with national Medicaid policy experts to confirm that no state or DC Medicaid program had a new prescription cap policy as of July 1, 2023.^20^ Phase 1 methodological details can be found in the Supplementary material.

### Phase 2: content analysis

#### Phase 2 data extraction

Phase 1 data from each policy source were systematically extracted and entered into a spreadsheet. The rows of the spreadsheet represented the 12 states with cap policies, and the columns contained information on the general cap policy (eg, number of prescriptions covered, prescriber and pharmacist override policies, exempt demographic groups and drug classes), and whether MOUD counts toward the cap. Exact policy language from state Medicaid websites, handbooks, and other online sources was also entered into the extraction spreadsheet, and the source of each data point was documented (see Supplementary material).

#### Phase 2 analysis

The extracted data were quantitatively and qualitatively analyzed. In instances where policy information conflicted across sources and steps (eg, online, expert survey, RFI), the most contemporary policy information was analyzed and reported.

The total number of state Medicaid programs operating a cap policy during the study period was calculated, along with the number of states that had MOUD-specific and demographic exemptions and pharmacy override stipulations.

The extracted policy data were narratively summarized according to general and MOUD-specific criteria, with attention given to cross-state policy similarities and differences. The narrative summaries were discussed with the full study team and used to contextualize the policy count data.

## Results

### Phase one: search process findings

Findings from the Phase 1 policy identification process and a detailed narrative summary of the results are available in the Supplementary material. Briefly, at the end of Phase 1, most of the cap policy information was identified for all states, although information on cap-specific pharmacy override policies and MOUD exemptions could not be identified for Florida.

### Phase two: content analysis findings

Table 1 provides details on the general and MOUD-specific cap policies by state. The most up-to-date information source identified in Phase 1, regardless of step, was used to populate Table 1 and conduct the content analysis.

**Table 1.** Findings of content analysis of state Medicaid prescription cap policies, 2023-2024.

	General					MOUD-specific
State	Maximum number of general Rx per month | brand-name cap	Maximum number of controlled substance Rx per month	Exempt enrollees	Exempt classes of drugs (not including MOUD)	Prescriber and pharmacist override exemptions	MOUD exemption
Alabama	5 Rx | 4 brand-name		<21 years of age Nursing home residents	Antipsychotics, antiretrovirals, and anti-epileptic Rx - up to 10 Three-month maintenance supply drugs	Yes	No
Arkansas	6 Rx | —		<21 years of age Enrollees in PASSE or ARHOME Assisted living residents up to 9 medically necessary Rx	High blood pressure, high cholesterol, bleeding disorders, diabetes, inhalers for breathing disorders, birth control pills, contraceptives, and medications that help you stop smoking Medicaid covered drugs for Medicaid-eligible long-term care facility residents or Medicaid-eligible clients <21 years of age in the Child Health Services/Early and Periodic Screening, Diagnosis, and Treatment Program	No	Yes
Florida	—	4 Rx controlled substances	Diagnosed with sickle cell or cancer	—	Unknown^a^	Unknown
Georgia	—	5 Rx narcotics	Diagnosed with cancer In hospice	Benzodiazepines	Yes	No
Illinois	4 Rx | 3 brand-name		<19 years of age Community integrated or supportive living residents	Oncolytics, antiretroviral agents, contraceptives, immunosuppressives, over-the-counter drugs, non-drug items such as blood glucose monitors and test strips, and antipsychotics	Yes	No
Kansas	4 non-preferred Rx | —		<21 years of age Diagnosed with cancer	Antiretroviral drugs, drugs on the Preferred Drug List, antirejection drugs used for transplant patients, state specified antiemetics and chemotherapy drugs, interferons, immune globulins, antihemophilic drugs, mental health drugs, and contraceptives	Yes	No
Louisiana	4 Rx | —		<21 years of age Diagnosed intellectual disability Pregnant Nursing homes or long-term care facility residents	Unknown	Yes	No
Mississippi	6 Rx | 2 non-preferred brand-name		Long-term care facility residents Early and Periodic Screening, Diagnosis and Treatment-eligible beneficiaries, if medically necessary	Clinician Administered Drugs and Implantable Drug System Devices dispensed by a pharmacy provider directly to a prescriber for administration	No	Yes (CADD only)
Oklahoma	6 Rx | 2 non-preferred brand-name		<21 years of age Diagnosed with cancer In hospice HCBS waiver program care plan enrollees up to 13 Rx	Antineoplastics, antiretroviral agents for persons diagnosed with AIDS or who have tested positive for HIV, frequently monitored prescription drugs, contraceptives, hemophilia drugs, compensable smoking and tobacco cessation products, opioid overdose reversal agents, certain carrier or diluent solutions used in compounds (ie, sodium chloride, sterile water, etc.), drugs used for the treatment of tuberculosis, and prenatal vitamins	No	Yes
Tennessee	5 Rx | 2 brand-name		<21 years of age Diagnosed with cancer or HIV Pregnant Intellectual disability facility residents Assisted living and nursing home residents Long-term care facility residents In hospice	Antianginals, antivirals, otics, antiarrhythmics, cardiac glycosides, pancreatic enzymes, antibiotics, diuretics, Parkinson's agents, anticoagulants, hyperkalemia agents, pheochromocytoma agents, anticonvulsants, hypotensives, potassium supplements (rx only), antidepressants, immune globulins, pulmonary arterial hypertension agents, antiemetics, multiple sclerosis agents, respiratory agents, antifungals, nitroglycerin preparations, rheumatoid arthritis agents, antiparasitics, ophthalmic preparations, thyroid hormones, antiplatelets, oral steroids, vasodilators, antipsychotics, oral thrombopoietic agents, vasopressors^a^	Yes	Yes
Texas	3 Rx | —		<21 years of age 21 years of age and older with acute pain HCBS waiver program enrollees	COVID-19 oral antivirals, COVID-19 test kits, COVID-19 vaccines, diabetic supplies, family planning products, home health supplies, mosquito repellents, influenza vaccines, opioids for acute pain, smoking cessation products	No	No
Wisconsin	—	5 Rx opioids	—	Liquid antitussive products containing opioids	Yes	Yes

Source: [Authors' analysis of publicly accessible Medicaid cap information, 2023-2024].

Abbreviations: Rx, prescriptions; — (none); HCBS, home and community based service.

Findings apply to Medicaid Fee-for-Service plans.

Unknown = contemporary (2023-2024) policy information not identified during Phase 1. MOUD exemption data not included in the 2019 KFF inventory.

^a^Unknown = contemporary (2023-2024) policy information not identified in Phase 1, but the 2019 KFF inventory indicated the presence of cap overrides.

As of October 1, 2020, the SUPPORT Act^21^ requires that all state Medicaid programs cover all forms of MOUD: methadone, buprenorphine, and naltrexone.

PASSE, Provider-led Arkansas Shared Savings Entity; ARHOME, Arkansas Health and Opportunity for Me Oklahoma exempt drugs: A complete list of the selected drugs considered as frequently monitored can be viewed on the agency's website at www.okhca.org.

#### General cap policies: summary of states with cap policies

Of the 12 states with cap policies, 9 had general prescription cap policies (ie, they were not specific to certain drug classes like narcotics). Among these states, the number of monthly covered prescriptions ranged from 3 in Texas to 6 in Arkansas, Mississippi, and Oklahoma. Five of these states had restrictions on the number of brand-name drugs covered, with brand-name caps ranging from 2 (Mississippi, Oklahoma, Tennessee) to 4 (Alabama) prescriptions per month. Three states had cap policies pertaining to controlled substances only: Florida (up to 4 controlled substances), Georgia (up to 5 narcotics), and Wisconsin (up to 3 opioids).

Of the 11 states for which pharmacy override exemption information could be identified, 7 were found to have such exemptions. Alabama, Kansas, and Louisiana override policies were dependent in whole or in part on a prescriber's documentation of the medical necessity of the medications exceeding the monthly cap. Prior authorization for pharmacy overrides was required in Illinois, Tennessee, and Wisconsin.

Of the 9 states with cap policies not specific to controlled substances, data on drug class exemptions were not identified for Louisiana in Phase 1, and 8 states had exemptions or a higher cap limit for certain drug types. The most common exempted drug class was contraceptives (*n* = 6 states), followed by antivirals (*n* = 6 states), particularly HIV antiretrovirals, antipsychotics (*n* = 3 states), and anti-hemophilic drugs (*n* = 3 states).

Nearly all states had cap exemptions for certain demographic groups. Cap policies applied to adult beneficiaries 21 years of age and older in Alabama, Arkansas, Kansas, Louisiana, Oklahoma, Tennessee, and Texas, and to beneficiaries 19 and older in Illinois. Beneficiaries with certain diagnoses were also exempt from the cap; the most common condition was cancer (5 states). Older and other individuals (eg, people with intellectual disabilities) living in long-term care facilities were also exempt from the cap in 5 states.

#### MOUD specific exemptions

Five of the 12 states had MOUD exemptions. Forms of MOUD that were covered by the state Medicaid program did not count toward the monthly prescription cap in Arkansas, Oklahoma, Tennessee, and Wisconsin. In Mississippi, Probuphine, Sublocade, and Vivitrol were listed as Clinician-Administered Drugs and Implantable Drug System Devices, which were exempt from the cap.

## Discussion

We conducted a multi-phase process to identify state Medicaid prescription cap policies and compared our findings of general and MOUD-specific policies across programs. We found that 12 states had prescription cap policies. Prescription caps ranged from 3 to 6 covered monthly prescriptions, with MOUD prescriptions counting toward the cap in 6 states. There was notable variation in the other drug classes and demographic groups exempt from cap policies across states. Findings have implications for Medicaid beneficiaries with OUD who might face difficulties finding information on the limits of their prescription drug coverage, as well as face barriers to receiving MOUD and other needed prescriptions if living in states where prescription caps operate.

We employed a three-step process to identify state cap policies operating between 2023 and 2024 and found that only 10 and 3 out of 12 states with active policies during this period had publicly accessible, up-to-date information online on general and MOUD-specific cap policies, respectively. In line with our goal of also assessing the ease of accessing this information, we found that most state Medicaid websites were difficult to navigate and contained limited up-to-date pharmacy coverage information. Further, the myriad of exemptions and stipulations of the policy caps rarely coalesced into a single document or website, and the language used in the online policy resources was highly technical. The difficulty of finding and deciphering policy information could inhibit beneficiaries, particularly those with low health literacy, from properly understanding their pharmacy benefits and appropriately navigating the cap.^22-26^ Additionally, our survey of state Medicaid experts and formal RFIs yielded incomplete and inaccurate information in some instances. The fact that the online search, survey, and both paid and unpaid formal RFIs did not necessarily yield accurate information on Medicaid policies further underscores the structural barriers that Medicaid beneficiaries and other members of the public face when trying to understand health insurance policies. Given these various barriers, it is recommended that states invest in efforts to make their policy information readily accessible online in easy-to-comprehend lay language (see the Wisconsin Medicaid website for an example).^27^ Such investments could serve to reduce the need for high-cost, state-funded Medicaid plan navigators. Further, Medicaid beneficiaries who are able to understand their pharmacy benefits and access medically necessary medications may be able to reduce adverse health outcomes that require higher-cost care, in turn resulting in cost savings for state Medicaid programs.^28,29^

Our content analysis of available Medicaid cap policy information found significant variability in the restrictiveness of the cap policies, in general and as they pertain to MOUD coverage. Drawing on the information identified through our Phase 1 process, Texas had the most restrictive policy of 3 prescriptions per month, with no exemptions for MOUD. Conversely, Wisconsin had the least restrictive policy, as the caps only pertained to opioid prescriptions, of which 5 prescriptions were covered per month, not including MOUD. For the states with general cap policies (ie, not specific to controlled substances), the average number of maximum covered prescriptions was 5, with all states having some cap exemptions based on a variety of enrollee characteristics–such as age (cap typically only applied to individuals 21 and older) or age-related characteristics (eg, resides in long-term care facility, in hospice, health conditions (eg, people with cancer); or specific drug classes (eg, antipsychotics, antiretrovirals, and MOUD). Seven states Medicaid programs also had policies allowing prescribers or pharmacists to override the prescription cap, typically when a prescriber provided documentation of medical necessity.

In total, of the 11 states with complete information on overrides and MOUD exemptions, Tennessee and Wisconsin had both; Alabama, Georgia, Illinois, Kansas, and Louisiana had overrides only; Arkansas, Mississippi, and Oklahoma had MOUD exemptions only; and Texas had neither. Although these policy carve-outs can provide greater access to MOUD when caps are in place, exemptions potentially pose fewer access barriers than overrides, as exemptions are typically automatic at the pharmacy point of sale and do not require a human to recognize the need for an override, place a request, or approve a request in order for a prescription to be filled.^20,30^ Given the persistence of the opioid and overdose epidemics,^31,32^ MOUD's essential role in reducing OUD-related morbidity and mortality,^3,4^ and the link between cap policies and fewer covered prescriptions for MOUD,^15,16^ state Medicaid programs should ensure that MOUD is exempt from cap policies to streamline access to these medications. Future longitudinal research should also seek to explore the impact of Medicaid cap policies on OUD-related morbidity and mortality for beneficiaries with OUD who are subject to the cap, and qualitative research should explore how prescribers, pharmacists, and beneficiaries with OUD navigate cap policies in real-world contexts.

It should be noted that Medicaid prescription caps are just one of several cost containment strategies that state Medicaid programs employ. Prior authorizations (PAs) requirements, another method through which state programs attempt to reduce spending, have been linked to reduced access to medically necessary care, including MOUD.^33-36^ Prior to January 2025, Alabama required prescribers to obtain a PA for buprenorphine before a beneficiary could receive a medication refill. According to Alabama Medicaid policies, PAs operate in tandem with Medicaid caps, as an approved PA for a prescription medication does not allow a beneficiary to receive more than the allowed number of prescriptions per month.^37^ In recognition of the structural barriers to accessing buprenorphine under the Medicaid program^38^ and potential for these medications to reduce fatal overdoses,^39^ Alabama revised its PA policy, enabling refills without a PA as of January 1, 2025, and new prescriptions without PA as of May 1, 2025, if certain other requirements were met. These revisions illustrate the ability of states to modify their Medicaid policies to maintain cost containment strategies for some medications, while simultaneously addressing the opioid epidemic through expanded access to MOUD.

## Limitations

This study had several limitations. To approximate the experience of a Medicaid beneficiary or another member of the general population seeking to identify pharmacy benefit information online, we intentionally engaged two analysts with limited pre-search knowledge of state Medicaid cap policies to conduct the Phase 1 online search. The analysts identified the search terms used and limited the online search to one hour per state, simulating the estimated time a beneficiary may spend searching for prescription drug coverage information online. Cap policy inventories known to the first and senior authors were restricted from our online search (eg, the 2019 KFF inventory). Additionally, we focused on policies pertaining to Medicaid fee-for-service, as there may be multiple managed care policies operating in each state, and only Mississippi Medicaid is known to require its managed care organizations to adhere to its prescription cap policies.^20^ Thus, the information identified in Phase 1 online search (Step 1) is unlikely to include all possible Medicaid policy information available online or fully simulate the experience of all Medicaid beneficiaries searching for cap policy information online.

In the Phase 1 survey, we relied on an American Medicaid Pharmacy Administrators Association (AMPAA) resource and a subsequent search for academic Medicaid experts in each state to identify individuals who could provide details on current Medicaid policies; this approach may have resulted in selection bias among survey respondents. Additionally, since we did not ask respondents to indicate their level of expertise regarding Medicaid cap policies, it is possible that there may have been lower levels of Medicaid program knowledge and expertise among survey respondents, which may have contributed to insufficient or inaccurate reporting of cap policy information via the survey. Additionally, we restricted the Phase 1 process to 12 months. It is possible that with more time, we may have received the requested information via the final RFI process. Further, although we employed a multi-pronged approach to determine that the remaining 37 states did not have prescription cap policies operating as of July 1, 2023,^20^ our approach did not include a national survey of Medicaid experts or a formal RFI. Thus, it is possible that one or more states may have implemented these policies or modified their existing policy in the time since the search was conducted. Finally, this study cannot be used to infer the effect of state Medicaid prescription cap policy characteristics on patient outcomes or the cost-effectiveness of implementing these policies.

## Conclusion

Limited contemporary information on general and MOUD-specific Medicaid prescription cap policies was publicly accessible online, which may pose a barrier to Medicaid beneficiaries with OUD and their providers, when seeking to understand prescription drug benefits. Additionally, MOUD was subject to prescription cap policies in most states that operated these policies, which may negatively impact access to medically necessary medications for low-income Medicaid beneficiaries with OUD and comorbid chronic conditions living in cap states. To ensure access to medically necessary medications such as MOUD, state Medicaid programs should invest in easy-to-navigate websites that include clear and up-to-date policy information on prescription drug coverage that can be readily accessed by beneficiaries, providers, and members of the general public. Qualitative and longitudinal quantitative studies exploring the impact of Medicaid prescription cap policies on OUD-related morbidity and mortality are also warranted.

## Supplementary Material

qxaf203_Supplementary_Data

## Data Availability

Data available upon request from the corresponding author.
