# Supplementary material for: Medicaid prescription cap policies and exemptions for medications for opioid use disorder: A process and content analysis
Source: Health Aff Sch. 2025 Oct 27;3(11):qxaf203. doi: 10.1093/haschl/qxaf203 (PMC12612678; doi:10.1093/haschl/qxaf203)
Supplement: qxaf203_Supplementary_Data [file qxaf203_supplementary_data.zip › Supplemental Materials_10.15.25.docx]

***Medicaid Prescription Cap Policies and Exemptions for Medications for Opioid Use Disorder: A Process and Content Analysis***

**Methods and Narrative Results**. Phase 1 Process Analysis.

**Table A1.** Results of Phase 1 state Medicaid cap policy identification process.

**Table A2.** Table A1 key.

**Data Sources.** Phase 2 Content Analysis.

**Phase 1 Methods**

Phase 1 - Step 1: Online Search

Two analysts with limited pre-search knowledge of Medicaid cap policies conducted the initial search. For each of the 12 states, the analysts conducted independent online searches for contemporary state resources on Medicaid pharmacy benefits (e.g., Medicaid program websites, Medicaid handbooks) using standardized search terms. Each analyst searched for resources containing core and supplemental information on both general (i.e., number of drugs included in the cap, exempt demographic groups and medications, pharmacy override policies) and MOUD-specific (i.e., MOUD exemptions) cap policies. Core information was used for the Phase 2 content analysis. Supplemental data included information that was initially being considered for inclusion in the analysis, but was ultimately excluded as it was not directly linked to cap policies (e.g., MOUD counseling requirements) or it was no longer of relevance due to the passage of the SUPPORT Act (i.e., types of MOUD covered). When official state resources (e.g., Medicaid website data, provider or pharmacy handbooks) could not be identified, third-party resources (e.g., policy coverage materials from the American Society of Addiction Medicine) were identified and used to determine coverage information. Prior to conducting the full search, the analysts each extracted details from a single-state search and then created a standardized search process that included key search terms. For each state, the analysts wrote memos narratively summarizing the online policy search process.

Phase 1 - Step 2: Survey of State Medicaid Policy Experts

Independent of the completeness of available online cap policy information, in the second step of Phase 1, state Medicaid policy experts completed a brief, 20-item survey about their state Medicaid program’s prescription cap policy and supplemental data of interest. Core survey items assessed the number of medications included in the state Medicaid prescription cap policy, including brand-name drugs, and whether there are pharmacy override policies and exemptions for specific conditions and drug classes, such as MOUD. Respondents were asked to upload or provide links to contemporary policy information. Using an American Medicaid Pharmacy Administrators Association (AMPAA) list of state Medicaid representatives, the analysts contacted experts via email or phone to complete the survey. When AMPAA-recommended experts were unresponsive or unable to answer the policy questions (i.e., Georgia, Florida), the analysts conducted Google Scholar and PubMed searches to identify academic experts who published state-specific Medicaid and MOUD-related policy research and contacted them to complete the survey. In Georgia, the initially identified academic expert referred the analysts to a colleague with greater expertise. Experts had the option of self-administering the survey online (Qualtrics) or completing the survey by phone. Providing state Medicaid policy information was optional; no compensation was provided to those who provided information on these public policies via the self- or analyst-administered survey.

Phase 1 - Step 3: Request for Information

Five states had missing cap policy information at the end of Step 2 (see Table A1): Alabama, Florida, Illinois, Mississippi, and Texas. To gather missing data, the analysts submitted a formal Request for Information (RFI) to each of the 5 states’ Medicaid administrative offices. The RFI applications were free for 3 states; Mississippi charged a $120 fee, and Alabama charged a $400 fee.

Phase 1 Quantitative Measures and Analysis by Step

At the end of Phase 1, each step of the process was scored according to the ease of identifying general and MOUD-specific cap policy information, and the *availability* and *accuracy* of the identified information by state.

*Step 1: Online Search*. The *ease* of finding general and MOUD-specific information was scored from 1 = Most sources included Medicaid website(s) or handbooks to 4 = Most sources included third-party resources. The *availability* of general and MOUD-specific cap policy information was scored from 1 = all information was available to 3 = no information was available. The *accuracy* of identified online information was scored from 1 = all the information was up-to-date to 4 = none of the information was up-to-date. The general and MOUD scores were averaged for each measure to produce a combined score, with lower scores representing greater ease, availability, and accuracy. The combined score for each measure was summed to create a summary process score for the step (theoretical range = 1-10), with 1 representing optimal ease, availability, and accuracy.

*Step 2: Survey*. For Step 2, ease, availability, and accuracy were considered simultaneously. The *ease* of identifying cap policy information via the survey was scored from 1= expert contacted, survey completed to 3 = 1-2 experts contacted, no survey completed. The *availability* of the cap policy information gathered via the survey ranged from 1 = all survey questions completed to 3 = no survey items completed. The accuracy of the policy data provided via the survey ranged from 1 = all the information was up-to-date to 4 = none of the information was up-to-date (or never received). The scores for each measure were summed to create a summary score (theoretical range = 1-9), with 1 representing optimal ease, availability, and accuracy.

*Step 3: RFI*. For the final step of Phase 1, ease, availability, and accuracy were considered simultaneously. The *ease* of obtaining the policy information via the RFI was scored from 1 = no barriers encountered, obtained information, to 3 = barriers encountered, did not obtain information. The *availability* of the information was scored according to 1 = received all needed policy information, to 3 = did not receive any of the needed policy information. The *accuracy* of the policy information provided was scored according to 1 = all information was up-to-date to date to 3 = none of the information provided was up-to-date (or never received). The scores for each measure were summed to create a summary score (theoretical range = 1-9), with 1 representing optimal ease, availability, and accuracy.

End of Phase 1. The c*ompleteness* of the core general and MOUD-specific cap policies and supplemental data was summarized by step and overall (see Table A1).

Phase 1 Qualitative Analysis

The Phase 1 narrative process analysis was restricted to the online search in order to characterize the experiences of members of the general population (e.g., Medicare beneficiaries) looking to identify pharmacy plan benefits through free and accessible online sources. First, drawing on thematic analysis methods,^[[1]](#footnote-1)^ the analysts open-coded their online search process memos for attributes of the online search process that would facilitate easy access to up-to-date pharmacy benefit information. The analysts then combined their individually identified attribute codes to create a unified list. Through a series of discussions with the analytic team, the list of attributes was synthesized into the following themes: availability of the state Medicaid website; ease of navigating the website; availability of an electronic Medicaid plan handbook containing pharmacy benefit information; and availability of date-stamped materials. Using a narrative synthesis approach,^21^ the attributes were used to contextualize the quantitative process data and qualitatively compare and contrast the state(s) with the most and least accessible and accurate online Medicaid cap policy information.

**Phase 1 Results**

Step 1- Online Search:

Of the 12 states included in the Step 1 online search, Wisconsin received the best summary score (4 out of 10) for the overall ease of identifying general and MOUD-specific information online and the availability and accuracy of the identified information. Florida and Georgia received the worst score (7.5 out of 10).

Ease: When states were scored according to the ease of identifying official state Medicaid websites, handbooks, or other policy documents, Alabama and Mississippi had the best score (1 out of 4), given that most of the sources identified were official state Medicaid resources, as opposed to third-party sources.

Availability: Louisiana and Wisconsin had the best availability score (1 out of 3), given that both general and MOUD-specific information were identified online. Florida received the worst score (2.5 out of 3), given that only some of the general policy information was available online, and no MOUD-specific policy information could be located online.

Accuracy: Up-to-date general and MOUD-specific information could only be identified for Arkansas and Wisconsin, resulting in the best scores (1 out of 3). Kansas received a score of 2.5 out of 3 (worst), given that some of the general cap policy information and all of the MOUD-specific information were out of date.

The narrative synthesis of the online search process identified attributes of online resources that would facilitate easy access to up-to-date information for Medicaid beneficiaries and others seeking to understand prescription drug coverage benefits across state Medicaid programs. An official state Medicaid policy source was considered to be accessible if: 1) information was available on a single website; 2) the Medicaid program had an electronically searchable Medicaid handbook; and 3) the resource listed the dates when it was created or updated. The Wisconsin Department of Health Services’ Medicaid website^[[2]](#footnote-2)^ is illustrative of these qualities, as it provides multiple relevant hyperlinks on a single landing page, the date for when the policy or documents were effective, and a hyperlink to an electronic pharmacy handbook that allows for specific information regarding prescription drug coverage (e.g., MOUD exclusion) to be searched and identified. Conversely, states were considered to be inaccessible for Medicaid beneficiaries seeking to utilize official state online resources to understand their Medicaid pharmacy benefits–if they: 1) lacked publicly accessible online information on the state Medicaid website or other official state electronic resources, or 2) had out-of-date online information.

Step 2: Survey of State Medicaid Policy Experts

Overall, Arkansas, Kansas, Louisiana, and Wisconsin scored the best (3 out of 9) regarding the ease of identifying an expert to answer the survey questions and the availability and accuracy of the general and MOUD-specific information received. Alabama, Florida, Illinois, Mississippi, and Texas scored the worst (9 out of 9).

Ease: Experts from every state responded to the survey invitation, except Alabama and Mississippi. Arkansas, Kansas, Louisiana, Oklahoma, Tennessee, and Wisconsin fared the best in terms of the ease of accessing expert-derived information (1 out of 3). Alabama, Florida, Illinois, Mississippi, and Texas scored the worst (3 out of 3), as no information was obtained from the experts.

Availability: Of the 10 state experts who responded to our survey requests, the respondent in Florida indicated “unsure” for all survey questions; the respondent in Illinois said they were unable to answer our questions; and the respondent in Texas indicated that they could not provide information on the policy due to pending state litigation, resulting in a score of 3 out of 3. Experts from the remaining 7 states completed survey information online or by phone, although the data provided by the Georgia respondent was incomplete, resulting in a score of 2 out of 3 for Georgia and a score of 1 out of 3 for the 6 states with complete data.

Accuracy: Although data was successfully collected from respondents in Arkansas, Georgia, Kansas, Louisiana, Oklahoma, Tennessee, and Wisconsin, some of the policy information provided by the respondents in Georgia was incomplete – resulting in worse score (2 out of 3) for these states, and better scores (1 out of 3) for Arkansas, Kansas, Louisiana, Oklahoma, Tennessee, and Wisconsin.

Step 3: Request for Information

A formal RFI was submitted for the 5 states with incomplete information at the end of Step 2: Alabama, Florida, Illinois, Mississippi, and Texas. Mississippi and Texas received the best overall RFI score (4 out of 9), followed by Illinois (6 out of 9), and Alabama and Florida (9 out of 9).

Difficulty: Alabama charged $400 to process the request, which was required to be submitted by an Alabama resident. Despite following the requirements and paying the fee, the information was never received, even after multiple follow-up attempts; this resulted in a score of 3 out of 3. Mississippi charged $120 to process the request, but subsequently provided the requested information, resulting in a lower score of 2 out of 3. Despite being informed by a Texas Medicaid representative that the state could not provide information on the policy via the survey due to an active lawsuit, we received a timely response following our RFI, resulting in a score of 1 out of 3.

Availability: No information was received from Alabama or Florida, resulting in a poor score of 3 out of 3. Comprehensive information was received from Mississippi, resulting in the best score (1 out of 3).

Accuracy: The information we received in Mississippi and Texas was up-to-date and provided some (Texas) or a lot of (Mississippi) additional information not found in the previous two steps (score 1 out of 3). The information received from Illinois was outdated and offered minimal MOUD-specific policy information (score 3 out of 3).

| **Table A1.** Results of Phase 1 state Medicaid cap policy identification process. | | | | | | |
| --- | --- | --- | --- | --- | --- | --- |
|  | **Step 1:  Online Search Scores** | | | **Step 2: Survey Scores** | **Step 3:  RFI Score** | **Completeness of Data -  End of Phase 1** |
|  | **General** | **MOUD** | **Combined** | **Combined** | **Combined** | **Combined** |
| **Alabama** | -- | -- | --- | --- | --- | Step 1: 2024 core policy data were readily available online.  Step 2: Expert did not complete the survey.  Step 3: A $400 RFI was submitted for supplemental data; the state never sent the requested information.  End of Phase 1: Core data complete; supplemental data incomplete. |
| Ease of identifying cap policy info | -- | -- | 1 | 3 | 3 |  |
| Availability of cap policy information | 2 | 1 | 1.5 | 3 | 3 |  |
| Accuracy of cap policy information | 1 | 3 | 2 | 3 | 3 |  |
| Summary score | --- | --- | **4.5** | **9** | **9** |  |
| **Arkansas** | --- | --- | --- | --- | --- | Step 1: 2024 core and supplemental policy data were readily available online.  Step 2: Expert confirmed Step 1 data.  Step 3: N/A  End of Phase 1: Core and supplemental data complete. |
| Ease of identifying cap policy info | -- | -- | 2 | 1 | --- |  |
| Availability of cap policy info | 2 | 1 | 1.5 | 1 | --- |  |
| Accuracy of cap policy info | 1 | 1 | 1 | 1 | --- |  |
| Summary score | --- | --- | **4.5** | **3** | --- |  |
| **Florida** | -- | -- | --- | --- | --- | Step 1: 2023 partial core and supplemental policy data were identified online, but somewhat difficult to find.  Step 2: Expert was unable to answer the survey questions.  Step 3: An RFI was submitted; data never received.  End of Phase 1: Core override and MOUD exemption data incomplete; supplemental data incomplete. |
| Ease of identifying cap policy info | -- | -- | 3 | 3 | 3 |  |
| Availability of cap policy info | 2 | 3 | 2.5 | 3 | 3 |  |
| Accuracy of cap policy info | 2 | 2 | 2 | 3 | 3 |  |
| Summary score | -- | -- | **7.5** | **9** | **9** |  |
| **Georgia** | -- | -- | --- | --- | --- | Step 1: 2024 core policy data were identified online, but difficult to find.  Step 2: Expert provided links to data but did not answer some questions.  Step 3: N/A  End of Phase 1: Core and supplemental data complete. |
| Ease of identifying cap policy info | -- | -- | 4 | 2 | --- |  |
| Availability of cap policy info | 2 | 2 | 2 | 2 | --- |  |
| Accuracy of cap policy info | 1 | 2 | 1.5 | 2 | --- |  |
| Summary score | -- | -- | **7.5** | **6** | --- |  |
| **Illinois** | -- | -- | --- | --- | --- | Step 1: 2024 core policy data were readily identified online.  Step 2: Expert unable to answer the survey questions.  Step 3: An RFI was submitted for supplemental data, but the data received were out of date.  End of Phase 1: Core data completed; supplemental data incomplete. |
| Ease of identifying cap policy info | -- | -- | 2 | 3 | 1 |  |
| Availability of cap policy info | 2 | 2 | 2 | 3 | 2 |  |
| Accuracy of cap policy info | 2 | 2 | 2 | 3 | 3 |  |
| Summary score | -- | -- | **6** | **9** | **6** |  |
| **Kansas** | -- | -- | --- | --- | --- | Step 1: 2024 core policy data were available online, but somewhat difficult to find.  Step 2: Expert confirmed core data and provided supplemental data.  Step 3: N/A  End of Phase 1: Core and supplemental data complete. |
| Ease of identifying cap policy info | -- | -- | 3 | 1 | --- |  |
| Availability of cap policy info | 2 | 1 | 1.5 | 1 | --- |  |
| Accuracy of cap policy info | 2 | 3 | 2.5 | 1 | --- |  |
| Summary score | -- | -- | **7** | **3** | --- |  |
| **Louisiana** | -- | -- | --- | --- | --- | Step 1: 2024 core policy data were available online, but somewhat difficult to find.  Step 2: Expert confirmed core data and provided supplemental data.  Step 3: N/A  End of Phase 1: Core and supplemental data complete. |
| Ease of identifying cap policy info | -- | -- | 3 | 1 | --- |  |
| Availability of cap policy info | 1 | 1 | 1 | 1 | --- |  |
| Accuracy of cap policy info | 1 | 3 | 2 | 1 | --- |  |
| Summary score | -- | -- | **6** | **3** | --- |  |
| **Mississippi** | -- | -- | --- | --- | --- | Step 1: 2020 core policy data were readily available online but out of date.  Step 2: Expert did not complete the survey.  Step 3: A $120 RFI was submitted, providing complete data.  End of Phase 1: Core and supplemental data complete. |
| Ease of identifying cap policy info | -- | -- | **1** | 3 | 2 |  |
| Availability of cap policy info | 2 | 2 | 2 | 3 | 1 |  |
| Accuracy of cap policy info | 2 | 2 | 2 | 3 | 1 |  |
| Summary score | -- | -- | **5** | **9** | **4** |  |
| **Oklahoma** | -- | -- | --- | --- | --- | Step 1: 2022 core policy data were fairly available online.  Step 2: Expert confirmed core data and provided supplemental data.  Step 3: N/A  End of Phase 1: Core and supplemental data complete. |
| Ease of identifying cap policy info | -- | -- | 2 | 1 | --- |  |
| Availability of cap policy info | 2 | 2 | 2 | 1 | --- |  |
| Accuracy of cap policy info | 2 | 1 | 1.5 | 1 | --- |  |
| Summary score | -- | -- | **5.5** | **3** | --- |  |
| **Tennessee** | -- | -- | --- | --- | --- | Step 1: 2023 core policy data were available online, but somewhat difficult to find.  Step 2: Expert confirmed core data and provided supplemental data.  Step 3: N/A  End of Phase 1: Core and supplemental data complete. |
| Ease of identifying cap policy info | -- | -- | 3 | 1 | --- |  |
| Availability of cap policy info | 2 | 2 | 2 | 1 | --- |  |
| Accuracy of cap policy info | 2 | 2 | 2 | 1 | --- |  |
| Summary score | -- | -- | **7** | **3** | --- |  |
| **Texas** | -- | -- | --- | --- | --- | Step 1: 2023 core policy data were available online, but somewhat difficult to find.  Step 2: Expert did not complete survey.  Step 3: RFI confirmed core data and provided supplemental data.  End of Phase 1: Core and supplemental data complete. |
| Ease of identifying cap policy info | -- | -- | 3 | 3 | 1 |  |
| Availability of cap policy info | 2 | 2 | 2 | 3 | 2 |  |
| Accuracy of cap policy info | 2 | 2 | 2 | 3 | 1 |  |
| Summary score | -- | -- | **7** | **9** | **4** |  |
| **Wisconsin** | -- | -- | --- | --- | --- | Step 1: 2024 core and supplemental policy data were readily available online.  Step 2: Expert confirmed Step 1 data.  Step 3: N/A  End of Phase 1: Core and supplemental data complete. |
| Ease of identifying cap policy info | -- | -- | 2 | 1 | --- |  |
| Availability of cap policy info | 1 | 1 | 1 | 1 | --- |  |
| Accuracy of cap policy info | 1 | 1 | 1 | 1 | --- |  |
| Summary score | -- | -- | **4** | **3** | --- |  |

***Note***. RFI Request for information. RFI only sought for states with incomplete information at the end of Step 2. MOUD=Medications for Opioid Use Disorder. Core data = policy information used for Phase 2 content analysis. Supplemental data = additional MOUD-related data of interest that were ultimately excluded from the content analysis (see Phase 1 Methods in Appendix). Details on the operationalization of the ease, availability, and accuracy of the measures are shown in the table key below and narratively described in the Appendix methods.

**Table A2.** Table A1 key.

|  | **Online** | **Survey** | **RFI** |
| --- | --- | --- | --- |
| *Difficulty identifying cap policy info* |  |  |  |
| 1 | Most sources included Medicaid website(s) or handbooks | 1 expert contacted & completed survey | No barriers encountered, info received |
| 2 | Most sources included Medicaid website(s), handbooks, and other official state resources | 2 experts contacted & completed survey | Some barriers encountered, info received |
| 3 | Even number of official state and third-party resources | 1-2 experts contacted; neither completed survey | No info received |
| 4 | Most sources included third party resources | --- | --- |
| *Availability of cap policy info* |  |  |  |
| 1 | All information was available | Expert completed all survey questions | Received all needed policy info |
| 2 | Some information was available | Expert completed some survey questions | Received some of the needed policy info |
| 3 | No information was available | Expert did not complete | Did not receive any needed policy info |
| *Accuracy of cap policy info* |  |  |  |
| 1 | All information was up-to-date | All info was up-to-date | All info was up-to-date |
| 2 | Some of the information was up-to-date | Some of the info was up-to-date or unknown | Some of the info was up-to-date or unknown |
| 3 | None of the information was up-to-date | None of the info was up-to-date or no info provided | None of the info was up-to-date or no info provided |

***Note***. RFI Request for information.

**Appendix of Data Sources.** Phase 2 Content Analysis.

The most contemporary core data were used to conduct the Phase 2 content analysis and populate Table 1 (main text). In most cases, the data were identified through online searches. In some cases, the information was confirmed by a state Medicaid expert via the survey. The formally filed requests for information (RFI) in Mississippi and Texas provided additional up-to-date information. The RFI data received for Illinois were outdated and not used for the content analysis. The RFI data for Alabama and Florida were not received. RFIs were not filed for the remaining states. The specific sources for the content analysis are listed below by state. Yes = data used for content analysis; No = data not used for content analysis; N/A = not applicable, no data received. Links are provided for the online data sources. Survey and RFI data are available upon request.

**Alabama:** Content Analysis Data Source - Online search: Yes | Survey: N/A | RFI: N/A

Online data sources:

- Provider Manual, 2024 (Pharmacy Benefits, Page 511: https://medicaid.alabama.gov/content/Gated/7.6.1G_Archived_Manuals/7.6.1.G_PM_January_2024_Bookmarked.pdf
- Prescription Limit FAQ, 2024 <https://medicaid.alabama.gov/documents/4.0_Programs/4.3_Pharmacy-DME/4.3.8_Rx_Limits/4.3.8_Prescription_Limit_FAQS_2-14-24.pdf>

**Arkansas:** Content Analysis Data Source - Online search: Yes | Survey: Yes | RFI: N/A

2024 policy information was identified online. The online data was confirmed and supplemented by the survey data received on 5/2/24.

Online data sources:

- Prescriber Manual, 2024: <https://humanservices.arkansas.gov/wp-content/uploads/PHARMACY-1-20up.doc>

**Florida:** Content Analysis Data Source - Online search: Yes | Survey: N/A | RFI: N/A

Online data sources:

- Pharmacy Handbook, 2023: <https://ahca.myflorida.com/content/download/22022/file/Summary_of_Drug_Limitations_2023-06-30_v137.pdf>

**Georgia:** Content Analysis Data Source - Online search: Yes | Survey: Yes | RFI: N/A

Online data sources:

- State Medicaid Website, 2024 🡪 Part II: Policies and Procedures for Pharmacy Services <https://www.mmis.georgia.gov/portal/PubAccess.Provider%20Information/Provider%20Manuals/tabId/18/Default.aspx>

**Illinois:** Content Analysis Data Source - Online search: Yes | Survey: N/A | RFI: No

Online data sources:

- State Medicaid Website, 2024: <https://hfs.illinois.gov/medicalproviders/pharmacy/fourprescriptionpolicy.html>

**Kansas:** Content Analysis Data Source - Online search: Yes | Survey: Yes | RFI: N/A

2024 policy information was identified online. The online data was confirmed and supplemented by the survey data received on 6/3/24.

Online data sources:

- Provider Manual, 2024: <https://portal.kmap-state-ks.us/Documents/Provider/Provider%20Manuals/Pharmacy_23327_23329.pdf>

**Louisiana:** Content Analysis Data Source - Online search: Yes | Survey: Yes | RFI: N/A

2023 policy information was identified online. The online data was confirmed and supplemented by the survey data received on 6/25/24.

Online data sources:

- Pharmacy Manual: <https://www.lamedicaid.com/provweb1/providermanuals/manuals/PHARMACY/PHARMACY.pdf>

**Mississippi:** Content Analysis Data Source - Online search: Yes | Survey: N/A | RFI: Yes

2020 policy information was identified online. The online data was confirmed and supplemented by the RFI data received on 6/14/24.

Online data sources:

- Pharmacy Handbook, 2020: <https://medicaid.ms.gov/wp-content/uploads/2020/07/Title-23-Part-214-Pharmacy-Services-eff.-08.01.20.pdf>

**Oklahoma:** Content Analysis Data Source - Online search: Yes | Survey: Yes | RFI: N/A

2022 policy information was identified online. The online data was confirmed and supplemented by the survey data received on 5/10/24.

- State Medicaid Website, 2022: <https://oklahoma.gov/ohca/individuals/mysoonercare/soonercare-benefits/prescriptions-drugs.html>

**Tennessee:** Content Analysis Data Source - Online search: Yes | Survey: Yes | RFI: N/A

2023 policy information was identified online. The online data was confirmed and supplemented by the survey data received on 5/13/24.

Online data sources:

- Pharmacy Manual, 2023: <https://contenthub-aem.optumrx.com/content/dam/contenthub/onboarding/assets/Tenncare/pharmacist/provider-manual/Division%20of%20TennCare%20Pharmacy%20Provider%20Manual.pdf>

**Texas:** Content Analysis Data Source - Online search: Yes | Survey: N/A | RFI: Yes

Online data sources:

- Provider Manual, 2023: <https://www.tmhp.com/sites/default/files/file-library/resources/provider-manuals/tmppm/pdf-chapters/2023/2023-08-august/2_15_Outpatient_Drug.pdf>

**Wisconsin:** Content Analysis Data Source - Online search: Yes | Survey: Yes | RFI: N/A

2024 policy information was identified online. The online data was confirmed and supplemented by the staff-administered survey received on 9/9/24.

Online data sources:

- State Medicaid Website, 2024: <https://www.forwardhealth.wi.gov/WIPortal/Subsystem/KW/Display.aspx?ia=1&p=1&sa=48&s=2&c=61&nt=Opioid+Monthly+Prescription+Fill+Limit&adv=Y>

1. Braun V, Clarke V: Using thematic analysis in psychology. Qual Res Psychol 2006;3:77-101. [↑](#footnote-ref-1)
2. Medicaid in Wisconsin. Wisconsin Department of Health Services, 2025. [↑](#footnote-ref-2)
